# Nanocarrier-mediated probiotic delivery: a systematic meta-analysis assessing the biological effects

**DOI:** 10.1038/s41598-023-50972-x

**Published:** 2024-01-05

**Authors:** Ramendra Pati Pandey, Gunjan, Himanshu, Riya Mukherjee, Chung-Ming Chang

**Affiliations:** 1 School of Health Sciences and Technology (SOHST), UPES, Dehradun, Uttarakhand 248007 India; 2 Graduate Institute of Biomedical Sciences, Chang Gung University, No. 259, Wenhua 1st Road, Guishan District, Taoyuan City, 33302 Taiwan (R.O.C.); 3 Master & Ph.D Program in Biotechnology Industry, Chang Gung University, No. 259, Wenhua 1st Road, Guishan District, Taoyuan City, 33302 Taiwan (R.O.C.); 4 Department of Medical Biotechnology and Laboratory Science, Chang Gung University, No. 259, Wenhua 1st Road, Guishan District, Taoyuan city 33302, Taiwan (R.O.C.)

**Keywords:** Biotechnology, Nanoscience and technology

## Abstract

Probiotics have gained a significant attention as a promising way to improve gut health and overall well-being. The increasing recognition of the potential health advantages associated with functional food products, leading to a specific emphasis on co-encapsulating probiotic bacteria and bioactive compounds within a unified matrix. To further explore this concept, a meta-analysis was performed to assess the effects of probiotics encapsulated in nanoparticles. A comprehensive meta-analysis was conducted, encompassing 10 papers published from 2017 to 2022, focusing on the encapsulation of probiotics within nanoparticles and their viability in various gastrointestinal conditions. The selection of these papers was based on their direct relevance to the research topic. Random-effect models were used to aggregate study-specific risk estimates. In the majority of studies, it was observed that nano-encapsulated nanoparticles showed improved viability over time compared to their free state counterparts. At various time intervals, the odds ratios (OR) with 95% confidence intervals (CI) were estimated using fixed and random effect models. At 0 min, the OR (95%CI) was 2.79 (2.79; 2.80) and 2.38 (2.14; 2.64) for. At 30 and 60 min observation was at similar rate of 2.23 (2.23; 2.24) and 2.05 (1.73; 2.43). However, at 90 min it was 1.39 (1.39; 1.39) and 1.66 (1.29; 2.14) and at 120 min 2.41 (2.41; 2.42) and 2.03 (1.63; 2.52). Overall evaluation of encapsulation revealed an improvement in probiotic bacterial viability in simulated the gastrointestinal environments.

## Introduction

Probiotics are nonpathogenic bacteria that are naturally derived from sources such as dairy foods. The global probiotics market is experiencing rapid expansion, and there is a growing momentum in research efforts to convert probiotics into medicinal adjuvants. Accordingly, the global probiotics market rose every year. The medical usage of probiotics has a lengthy history, grounded in the concept that oral or topical probiotic treatment has the potential to replace damaged human microbiota. Additionally, they have been linked to a variety of favorable outcomes through aiding digestion^1,2^, increasing nutrient absorption^3,4^, improve metabolism (including lactose intolerance^5^, calcium absorption^6^), and strengthening the immune system^7^. It also has a regulatory function in the body, such as boosting biological defense systems, reducing particular diseases, managing mental and physical illnesses, and slowing the aging process^8^. Ensuring the adequate quantity and targeted release of probiotics is crucial for their effective delivery to the large intestine. This is because free probiotics are susceptible to destruction due to the harsh conditions present in the upper gastrointestinal tract (GIT) of humans. These conditions include antimicrobial lysozyme in the oral cavity^9^ acidic environment in the gut^10^ bile salts and digestive enzymes in the small intestine^11^, as well as other factors like osmotic pressure and oxidative stress across the gastrointestinal tract.

Presently, microbial strains need to fulfill specific criteria to be recognized as potential probiotics. In accordance with the guidelines proposed by the FAO/WHO, each probiotic strain must undergo accurate identification and undergo various in vitro assays to evaluate their functional properties^12^. Despite the availability of numerous commercial probiotics in the market, there continues to be an ongoing need for new probiotic strains that exhibit improved characteristics compared to current ones. In recent years, several bacterial species including *Lactobacillus* spp. have emerged as potential probiotics. *Lactobacilli* are a type of Gram-positive bacteria that are catalase-negative^13^. They are naturally present in the oral cavity, intestine, and female vaginal tract. They play a crucial function in controlling the growth of undesirable microorganisms, making them natural bio preservatives^14^. *Lactobacilli* have been designated as 'generally recognized as safe' (GRAS) by the United States Food and Drug Administration (USFDA) and 'qualified presumption of safety' (QPS) by the European Food Safety Authority (EFSA), allowing them to be used in food preparation^15^. Their significant economic importance has led to extensive research, resulting in a comprehensive understanding of their genomics and relationships with humans concerning both health and disease. *Lactobacillus* species are great candidates for probiotics due to these properties. The link between *Lactobacilli* and humans is mutually beneficial. *Lactobacillus* species aid the host in the digestion of specific dietary components and provide protection against infections^16^. Furthermore, continuing research is examining novel approaches to optimizing probiotic delivery, functionality, and monitoring through the use of new technologies such as nanotechnology^17^.

Nanotechnology contributes to various domains within the realms of science and technology. To get the beneficial effects, nanotechnology emerging as an effective alternative to traditional therapies. The convergence of probiotic science, with the realm of nanotechnology gives rise to a novel field called “nanoprobiotics”^18^. This evolving field employs a specific strategy to address certain limitations associated with the utilization of probiotics in food and therapeutic applications. It involves encapsulating probiotics and other bioactive components within protective shells of nanoparticles, which act as physical barriers. This technique aims to enhance the viability and bioavailability of probiotics by safeguarding them during storage and transit. This meta-analysis and systematic review present a comprehensive overview of recent nano-formulation approaches aimed at optimizing the delivery of probiotics, and formulation technologies utilized in the field to improve the efficacy and viability of nanoparticle encapsulated probiotics.

## Materials and methods

### Study design and search strategy

This study followed the guidelines set forth in the Preferred Reporting Items for Systematic Reviews and Meta-Analysis (PRISMA) statement in both its design and reporting^19^ Various Medline search engines, including PubMed, Google Scholar, Science Direct, Scopus and Web of Science were utilized to conduct a comprehensive review of the literature from 2016 to 2022, without language restrictions. Different MeSH terms (“probiotics” [MeSH Terms] OR “probiotics” [All Fields]) AND “Nanoparticle encapsulation” [MeSH Terms] OR “Nanoparticle encapsulation” [All Fields] AND “encapsulation techniques”) OR “probiotics” [MESH Terms] AND “Nano formulations” [All Fields] OR “probiotics” (MeSH Terms] AND “Nano formulations” [All Fields] OR “*Lactobacillus”* [MeSH Terms] AND “Nano formulations” [All Fields]) searched on different Medline databases and additional searches were conducted using known probiotic types, referencing author names, meeting abstracts, and the reference lists of included literature. Furthermore, a systematic search was conducted by reviewing the bibliographies of all publications obtained.

### Inclusion and exclusion criteria

We searched for the articles for the meta-analysis, the studies that were included met the following criteria: (1) articles should be from 2017 to 2022 (2) articles had to be published in peer-reviewed journals, (3) the probiotic bacteria used had to be *Lactobacillus* spp., (4) the articles needed to mention nanoparticle encapsulation materials and techniques, (5) only probiotics that met the standard criteria (living microbe, adequate dose, and demonstrated efficacy for optimal health effect) were considered, and (6) studies reporting survivability/viability outcomes in colony-forming units (CFU).

Whereas the following criteria were applied to exclude the articles (1) conference, abstracts, perspectives, review articles, and meta-analysis (2) study protocols and articles lacking full text or not published in English (3) articles that only mentioned microencapsulation but not nanoparticle-based encapsulation, and (4) studies that did not provide the necessary data.

### Data extraction

The data related to different types of nanoparticles, probiotics, and their encapsulation were collected and organized in a single sheet using Microsoft Office Excel® (2013). Prior to the complete extraction, a pre-test was conducted. The extracted data consisted of authors' names, publication years (2017–2022), microorganism species, encapsulating nanomaterials, encapsulating techniques, characteristics (with emphasis on particle size and morphological characteristics), viability, and encapsulation yield. The data underwent careful examination, and information specifically related to Nanocarrier-Mediated Probiotic Delivery was visualized in a table and forest plot, including the relevant citations, using Mendeley (version 1.19.8).

### Statistical analysis

The statistical analysis and generation of forest plots for pooled summary estimates were conducted using the meta or metafor package in R software^20^. The summary estimates were derived from pooled data of forest plots representing different time points after encapsulation of probiotics of the same type of probiotic bacteria (i.e., *Lactobacillus* spp.) and measuring the common outcome of probiotic bacteria survivability in the nanoparticles. 95% confidence intervals (CI) and odds ratios (OR) for both fixed-effect and random-effects models were calculated. The I^2^ and τ^2^ statistics were used to evaluate the heterogeneity of the data. All meta-analyses used random-effects models, and the results were displayed in forest plots. Furthermore, the statistical significance was validated by the *p*-value (*p* < 0.05).

### Publication bias

The assessment of publication bias is a vital step in ensuring the robustness and validity of our meta-analysis of the effects of probiotics encapsulated in nanoparticles. To evaluate the potential impact of publication bias on our findings, we employed several methods recommended in the field. We visually inspected risk of bias graph to detect asymmetry, a potential indicator of publication bias. By employing this comprehensive approaches, we aimed to account for any potential bias and ensure that our meta-analysis provides an unbiased synthesis of the available evidence on the efficacy of probiotics encapsulated in nanoparticles.

## Result

### Study selection and characteristics

**Figure 1:**
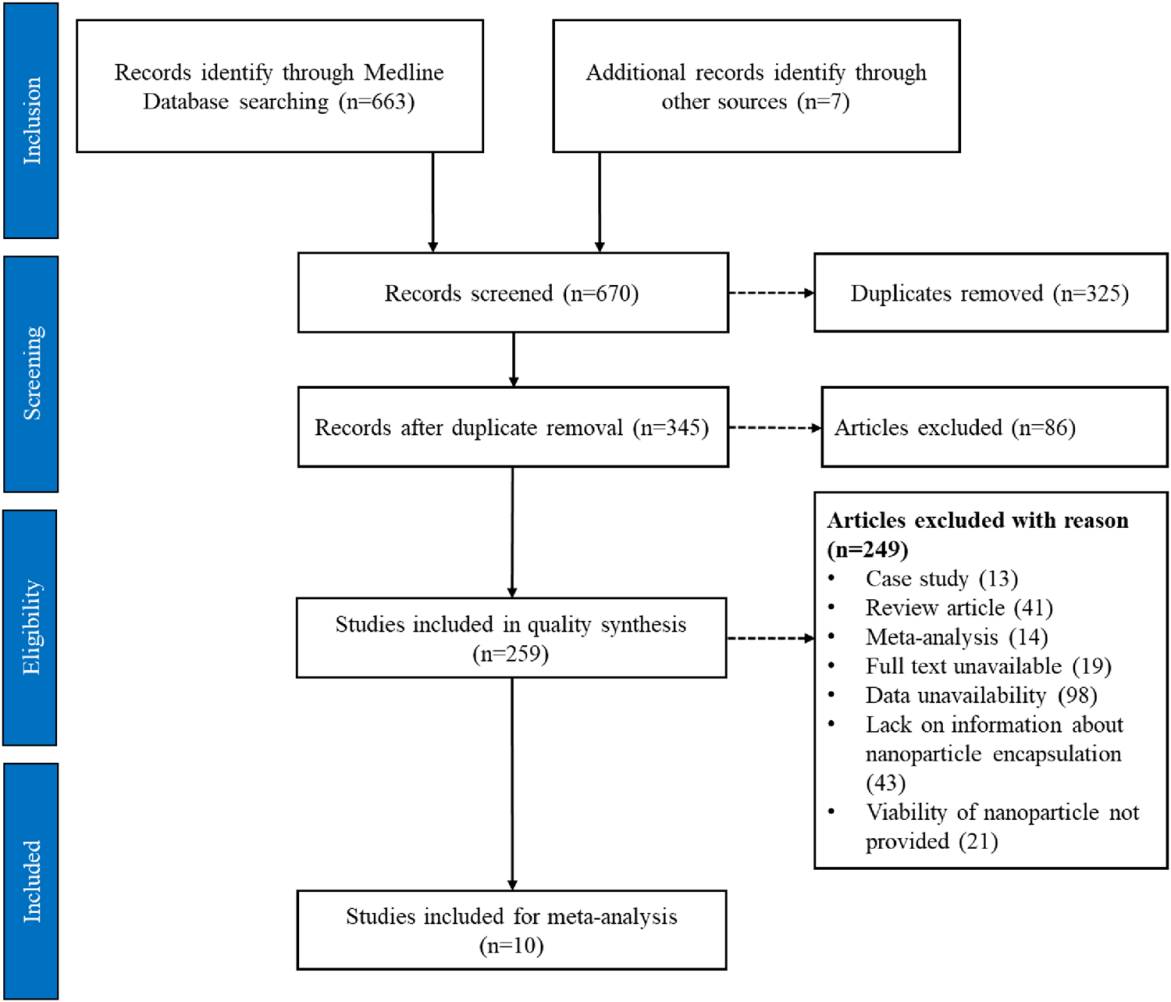
Visual Representation of the Study Selection Process using PRISMA Guidelines.

A total of 670 papers published between 2017 and 2022 were categorized using Medline databases. After removing duplicates, 345 studies underwent initial screening based on their title and abstract. From these, 86 articles were excluded due to irrelevance or redundancy, leaving 259 articles for further examination. Subsequently, 249 articles were excluded for various reasons: 13 were case studies, 41 were review articles, and 14 were meta-analyses. Additionally, 19 articles lacked full-text availability, and 98 articles lacked essential data or statistics. Furthermore, 43 articles did not provide information about nanoparticle encapsulation, and 21 articles did not report nanoparticle viability within the specified time frame. Ultimately, this meta-analysis and systematic review incorporated a total of 10 studies. The study selection process adhered to the PRISMA flow diagram, depicted in Fig. 1.

### Meta-analysis

#### Risk of bias

**Figure 2:**
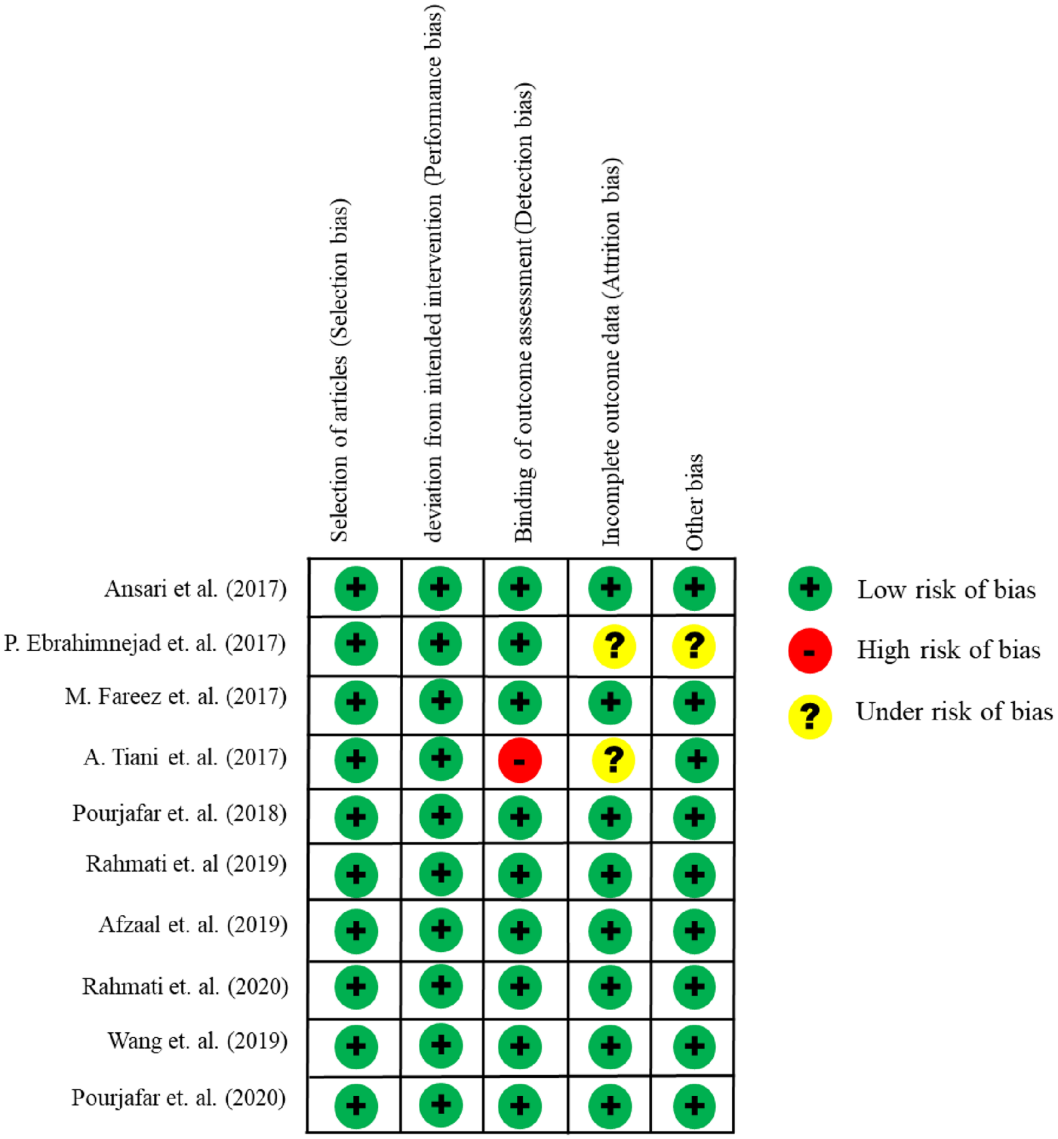
Risk of bias graph for the selected studies.

Figure 2 presents a comprehensive assessment of the overall risk and individual biases in each included study. All researchers conducted evaluations to determine the likelihood of bias, and the assessment findings demonstrated remarkable consistency across all investigations. On the basis of the outcomes displayed in Figure 2, it is clear that the study was conducted by by Tiani et al.^21^ exhibited a high risk of bias Furthermore, Ebrahimnejad et al.^22^ found a potential risk of bias in their investigation. This robust and uniform approach strengthens the reliability of the research paper's results, ensuring a high level of confidence in the reported biases and their impact on the study outcomes.

#### Literature data search and data mining

The meta-analysis process involved the selection of ten peer-reviewed research articles^21–30^ published between 2017 and 2021. These articles provided data on various aspects, including the study and publication year, probiotic species, encapsulating nanomaterials, encapsulating techniques, particle size, morphological characteristics, viability at different time intervals (0 min, 30 min, 60 min, 90 min, and 120 min), and encapsulation yield as illustrated in Table 1.

**Table 1:** Summary of subgroup analysis results.

Study (year)	Probiotic species	Encapsulating nanomaterials	Encapsulating technique	Characteristics of microcapsules		Viability (CFU/ml)						Encapsulation yield
				Particle size	Morphological characteristics	State	0 min	30 min	60 min	90 min	120 min	
Ansari et al. (2017)^24^	*Lactobacillus acidophilus*	Eudragit S100 and chitosan	Extrusion	100 nm	- 123.66 ± 41.73 μm in diameter - Sphere in shape - Polydispersity index was 0.410	Free	9.78	7.27	5.53	4.56	3.0	- Survivability increased
						Encapsulated	9.52	8.17	7.54	6.6	5.02	
Ebrahimnejad et al. (2017)^22^	*Lactobacillus acidophilus*	Chitosan	Ionic gelation	120 to 338 nm	- Spherical, distinct and regular shape of particle	Free	3.3	3.26	3.2	3.11	3.09	- Bacterial survival in simulated gastric and intestinal environments improves - Concentration of chitosan determine size of particles
						Encapsulated	3.2	3.27	3.25	3.23	3.23	
M. Fareez et al. (2017)^25^	*Lactobacillus plantarum*	Alginate	Extrusion-dripping	1299 to 1341 μm	- Beads appeared ‘crumpled’ and irregular in shape - Polydispersity index was 0.05	Free	10.4	9.4	7.5	3.6	3.15	- Probiotic bacteria show improved viability under simulated gastric and intestinal conditions - Concentration doesn’t determine size of particles
						Encapsulated	10.6	8.2	8.1	8.05	7.5	
A. Tiani et al. (2017)^21^	*Lactobacillus plantarum*	Sodium alginate	Extrusion	324 and 327 μm	- Irregularly shaped, 150–350 μm in diameter	Free	9.86	8.64	8.52	8.51	8.35	- Alginate micro beads maintained cell viability during refrigerated storage and enhanced resistance to simulated gastric and intestinal conditions
						Encapsulated	10.1	8.69	9.2	9.27	9.26	
Pourjafar et al. (2018)^26^	*Lactobacillus acidophilus*	Eudragit S100	Extrusion	100 nm	–	Free	10.5	8.0	6.1	5.4	8.35	- Probiotic bacteria survive in adverse conditions
						Encapsulated	11.1	9.1	8.3	6.5	8.35	
Rahmati et. al (2019)^29^	*Lactobacillus casei*	Eudragit S100	Homogenization/Supercritical antisolvent - technique	100 to 170 nm/120 nm	- 70–180 μm in diameter	Free	6.0	4.4	6.1	–	5.3	- Organoleptic attributes like texture, flavor, and aroma is improved - Function in gastric conditions improved
						Encapsulated	6.9	3.9	5.9	–	3.6	
Afzaal et al. (2019)^30^	*Lactobacillus casei*	Calcium alginate and whey protein	Extrusion	–	- 716–727 μm in diameter	Free	10.79	4.4	8.1	6.8	5.48	- Function is improved under simulated gastrointestinal conditions
						Encapsulated	10.72	9.8	9	6.3	7.65	
Rahmati et al. (2020)^28^	*Lactobacillus acidophilus*	Alginate chitosan, - Eudragit S100	Homogenization/Supercritical antisolvent - technique	110–170 nm/122 nm	- Polydispersity index was 0.460 - 80–180 μm in diameter	Free	6.4	6.4	5.0	–	4.3	- Viability increased
						Encapsulated	6.8	6.8	6.1	–	5.6	
Wang et al. (2019)^23^	*Lactobacillus pentosus*	Chitosan and sodium phytate	Layer-by-layer	–	- Zeta potential of coated nanoparticle is + 39.9 mV	Free	8.5	8.4	8.6	8.4	7.9	- Higher survival rates in simulated gastrointestinal fluid and bile salts
						Encapsulated	8.6	8.5	8.6	8.5	8	
Pourjafar et al. (2020)^27^	*Lactobacillus acidophilus*	Eudragit S100 and chitosan	Homogenization/Supercritical antisolvent - technique	100–150 nm/100 nm	- Polydispersity index was 0.410 - beads were sphere-shaped with - a mean diameter about 1 mm	Free	5.3	3.3	7.8	1.3	1.1	- Survivability increased
						Encapsulated	8.7	2.9	5.1	4.9	8	

#### Lactobacillus acidophilus

Five articles^22,24,26–28^ investigated the encapsulation of *Lactobacillus acidophilus*, and the majority of them employed Eudragit S100 nanomaterial in combination with chitosan^24,26,27,29^. However, one article by Ebrahimnejad et al.^22^ used chitosan alone as the encapsulating nanomaterial The encapsulation techniques varied among the studies, with Ansari et al^24^ and Pourjafar et al^26^ employing the extrusion method, Rahmati et al^29^ and Pourjafar et al^27^ using a different technique, and Ebrahimnejad et al^22^ utilizing the Ionic gelation method The average nanoparticle size across all the articles was observed to be 100 nm (Table 1).

#### Lactobacillus casei

In two separate studies^29,30^, *Lactobacillus casei* was the subject of investigation. Afzaal et al^30^ employed the extrusion technique for encapsulation, while Rahmati et al^29^ used Eudragit S100 nanomaterial with two distinct encapsulation techniques: homogenization and supercritical antisolvent The resulting nanoparticle sizes showed variability, with Rahmati et al^29^ achieving sizes ranging from 100 to 170 nm and 120 nm (as shown in Table 1) The encapsulation of *Lactobacillus casei* using different methods and nanomaterials demonstrates the versatility of the approach, allowing for control over the size of the nanoparticles.

#### Lactobacillus plantarum

Fareez et al^25^ and Tiani et al^21^ conducted separate studies involving *Lactobacillus plantarum* Both groups utilized alginate as the encapsulating material, with the extrusion technique employed in both cases However, notable differences in the size of the resulting nanoparticles were observed Fareez et al^25^ achieved nanoparticle sizes ranging from 1299 to 1341 μm, whereas Tiani et al^21^ obtained sizes of 324 and 327 μm Importantly, the alginate microbeads were found to effectively preserve the viability of the encapsulated cells during refrigerated storage. Additionally, these microbeads exhibited enhanced resistance to simulated gastric and intestinal conditions, suggesting their potential as protective carriers for *Lactobacillus plantarum* (Table 1).

#### Lactobacillus pentosus

In a study carried out by Wang et al^23^ *Lactobacillus pentosus* was encapsulated using chitosan and sodium phytate through a layer-by-layer approach. While the specific size of the nanoparticles was not mentioned in the article, the encapsulated nanoparticles exhibited higher survival rates when subjected to simulated gastrointestinal fluid and bile salts, as indicated in Table 1.

#### Analysis of nanoparticle encapsulated probiotic efficiency

In this study, a total of ten research studies^21–30^ were selected based on their suitability for quantitative analysis. The objective was to investigate the flexural strength data for different fraction weights of encapsulated probiotics in nanoparticles To analyze the data, five meta-analysis were conducted, and forest plots were constructed to visualize the results.

**Figure 3:**
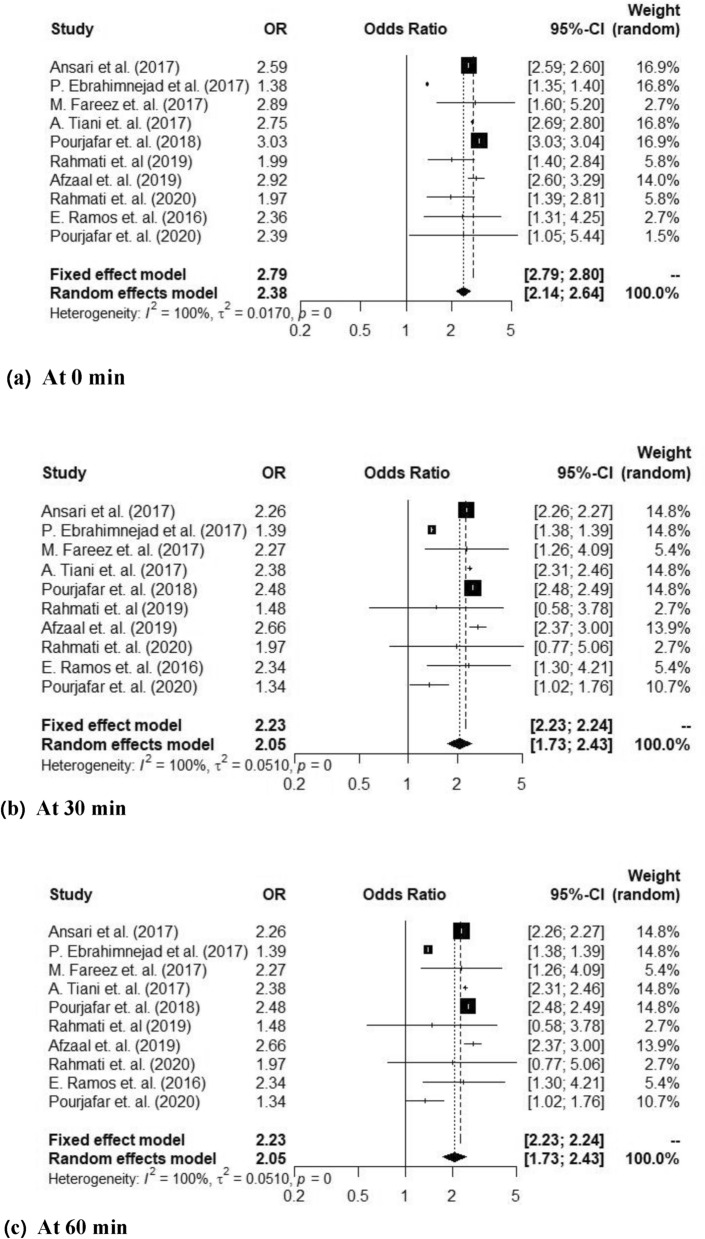

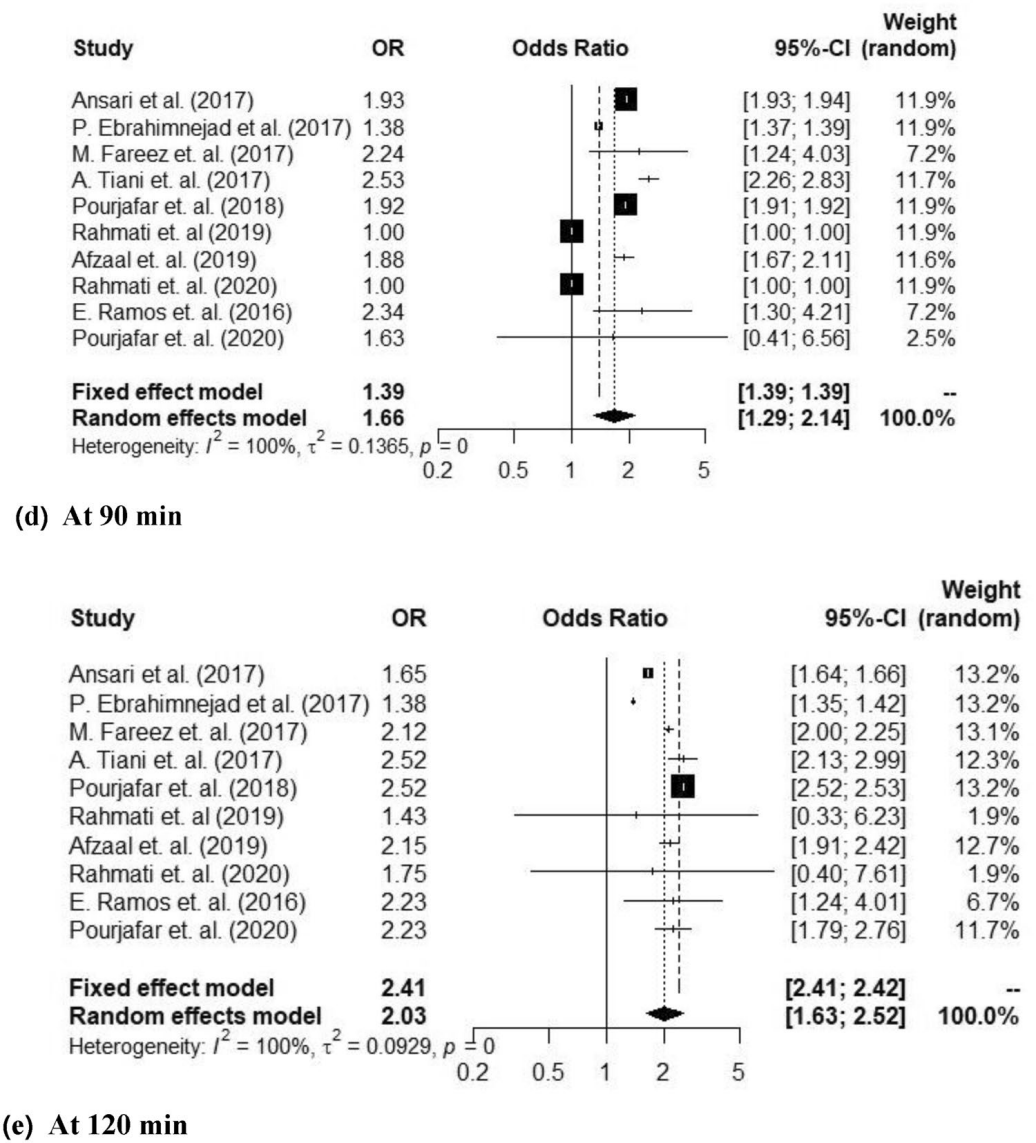
Forest plot of the viability of nanoparticle encapsulated probiotic *lactobacillus* spp showing the odd ratio (OR), 95% confidence intervals (CI) and weight (random) at different time intervals.

In Fig. 3a, the forest plot presents the results for the 0-min time point following lactobacillus spp. encapsulation, showing a 95% confidence interval (CI) of (2.79; 2.80) for the fixed effect model and (2.14; 2.64) for the random effect model. Notably, all included studies demonstrated a high level of heterogeneity with an inconsistency test (I^2^) of 100% and a study variance (τ^2^) for random-effects across studies of 0.0.0170. The odds ratio (OR) values were 2.79 for the fixed effect model and 2.38 for the random effect model, and the *p*-value of 0 indicates significant statistical significance at 0 min after encapsulation. Moving to Fig. 3b, representing the 30-min time point after encapsulation, the analysis yielded a 95% CI of (0.23; 2.24) for the fixed effect model and (1.73; 2.24) for the random effect model, with OR values of 2.23 and 2.05, respectively The included studies exhibited a low level of heterogeneity (I^2^) of 100% and a study variance (τ^2^) of 0.01510, with a p-value of 0 indicating strong statistical significance at 30 min. Similarly, Fig. 3c focused on the 60-min time point, revealing a 95% CI of (2.23; 2.24) for the fixed effect model and (1.73; 2.43) for the random effect model, with OR values of 2.23 and 2.05, and a low level of heterogeneity (I^2^) and study variance (τ^2^) of 0, and the *p*-value was 0.97 Figure 3d displayed the forest plot for the 90-min time point after encapsulation, with a 95% CI of (1.39; 1.39) for the fixed effect model and (1.29; 2.14) for the random effect model, OR values of 1.39 and 1.66, and a low level of heterogeneity (I^2^) of 100% and study variance (τ^2^) of 0.1365, while the *p*-value was 0 indicating statistical significance at 90 min Finally, Fig. 3e illustrated the forest plot for the 120-min time point after encapsulation, revealing a 95% CI of (2.41; 2.42) for the fixed effect model and (1.63; 2.52) for the random effect model, with OR values of 2.41 and 1.78, and a low level of heterogeneity (I^2^) of 2.41 and study variance (τ^2^) of 2.03, while the p-value was 0 indicating statistical significance at 120 min.

### Global market share of probiotics

The global food probiotics market recorded a significant milestone in 2022, reaching a value of US$ 60.5 billion. Forecasts indicate that this market is poised for further growth, with an estimated value of US$ 100.1 billion projected by 2030. This predicted growth implies a compound annual growth rate (CAGR) of 6.5% from 2023 to 2030. Notably, the Regulatory Affairs Professionals Society revealed that the total probiotic market surpassed US$ 48 billion in 2021, underlining its substantial presence. Among the geographical regions, North America stands out as the fastest-growing market in the food probiotics industry^31^. As consumers become increasingly aware of the potential health benefits associated with probiotic consumption, the market continues to expand, driven by factors like increased demand for functional foods and dietary supplements. The promising trajectory of the food probiotics market underscores its significance in the global food industry and its potential to provide opportunities for businesses operating in this sector. In terms of revenue, *Lactobacillus* held about 65% market share of the worldwide probiotics market in 2021, followed by *Streptococcus* and *Bifidobacterium*, in that order^32^. Due to the rising demand for non-pharmacological treatments to lower the cost of production for human probiotic applications, the market for *Lactobacilli*-based probiotics the estimated value of probiotics was worth more than USD 1.8 billion in 2022^33^.

## Discussion

This systematic review and meta-analysis provide the most in-depth look to date regarding the utilization of encapsulated probiotic viability efficacy. We have evaluated a total of 10 research papers related to probiotic encapsulation with nanoparticles and their efficacy that were published in the period between 2017 and 2022. A designed follow-up probiotics risk group assessment was performed at a variety of nanoparticles that is used as an encapsulating material for probiotic bacteria *Lactobacillus* spp. The forest plots provide an in-depth analysis of the data, showing that the I^2^ value of 100% shows that all observed variance is caused by heterogeneity rather than chance. In a random-effects meta-analysis model, the numbers also show the estimated between-study variance. Its range, 0.0929–0.02296, indicates that effect sizes among the research included in the study varied. According to this number, there is some variation in the impact sizes among studies, but the level of heterogeneity is not particularly high. The results of the current study concur that the statistically significant difference should be *p* 0.05, however this follow-up investigation with the forest plot shows the *p*-value is 0, which suggests that the observed data is statistically significant.

Despite the current limitations, the growing significance of new technologies and advancements in research on the impact of probiotics and postbiotics on human health based on the microbiota will undoubtedly be pivotal in crafting personalized treatments for prevalent diseases. The encapsulation of probiotics in nanoparticle provides numerous benefits for probiotics, such as enhancing their survival by shielding them from the severe circumstances of the gastrointestinal environment as well as external factors like oxygen, temperature, and light during storage and handling^34^. Additionally, it facilitates the precise and regulated release of the encapsulated materials at the appropriate concentrations within the digestive tract and makes it possible to incorporate probiotics at a range of concentration, ranging from lower to higher concentrations. These advantages underscore the potential of encapsulation as a significant approach, ranging from low to high levels. These advantages highlight the potential of encapsulation as a valuable technique for improving the functionality and effectiveness of probiotic products. However, until now, there have no standard use of nanoparticle encapsulated drug in humans were registered. Additionally the limited of research in this area offers researchers ample opportunities to explore and develop functional food products. By incorporating bioactive compounds and probiotics as co-encapsulation materials, researchers can create products that offer multiple functionalities and improve the delivery of active ingredients in the human gut, this presents a promising avenue for the development of innovative food formulations that provide enhanced health benefits and targeted effects on the human body^35^. The main drawbacks of adding natural food antimicrobials directly to food products could be eliminated by encapsulating them using various techniques.

Currently, probiotics are categorized and regulated differently by various regulatory agencies worldwide, including biologics, drugs, foods, and nutritional supplements. Consequently, each regulatory category has its own set of guidelines. To ensure the integrity, security, durability, and efficacy of probiotic formulations throughout the whole production, handling, storage chain, and post-marketing surveillance, it is crucial to establish an effective regulatory framework and harmonize guidelines. However, the lack of proper standardization parameters for probiotics poses a significant challenge in establishing the credibility of their health-promoting functions^36^. Different organizations in worldwide are working in order to build guidelines, policies and regulations. Several international organizations such as Food and Agriculture Organization (FAO)/ World health organization (WHO) established protocols for the assessment of probiotics in food products^12,16^. International dairy federation, initiated the formulation of protocols to assess distinct functional and safety attributes outlined in the FAO criteria for assessing probiotics in food^37^. Codex standard for fermented milk (CODEX STAN 243-2003), outlines the minimum quantities of characterizing and extra labeled microorganisms in yoghurt, acidophilus milk, kefir, kumis, and other fermented milks, in addition to other composition requirements^38^. International scientific association for probiotics and prebiotics, investigate the validation of techniques and the establishment of laboratory facilities for the analysis of microbiological content in probiotic products^39^ and World Gastroenterology Organization (WGO) focus on determining the genus, species, and strain of each probiotic present in a product, as well as the viability and survival rate of the probiotic strains throughout the product's shelf life^40^ as depicts in Table 2.

**Table 2:** Worldwide regulation and policies for probiotics products.

Organization	Role	References
Food and Agriculture Organization (FAO)/ World health organization (WHO)	Developed guidelines for evaluating probiotics in food	^12,16^
International dairy federation	Has begun developing procedures for determining specific functional and safety features indicated in the FAO recommendations for the evaluation of probiotics in food	^37^
Codex standard for fermented milk (CODEX STAN 243–2003)	This standard specifies minimum numbers of characterizing and extra labelled microorganisms in yoghurt, acidophilus milk, kefir, kumis, and other fermented milks, among other composition requirements	^38^
International scientific association for probiotics and prebiotics	The Industry Advisory Committee and the Board of Directors will explore technique validation and the development of laboratory locations to analyze probiotic product microbiological content	^39^
World Gastroenterology Organization (WGO)	It focus on the genus, species, and strain of each probiotic in a product, as well as the amount of viable cells of each probiotics strain that will survive till the end of the shelf-life	^40^

## Conclusion

With the ongoing expansion of the probiotic sector, an increasing number of individuals are recognizing the benefits that probiotics offer to human health. Probiotics play a crucial role in sustaining digestive health and addressing dysbiosis in intestinal flora. Moreover, they serve as preventive and therapeutic measures against various diseases such as obesity, colitis, colorectal cancer, and metabolic disorders. Consequently, the global probiotic market experiences continuous annual growth. Additionally, there are many benefits to entrapping probiotics in a nano system, including maintaining probiotic stability, delivering a barrier to protect them from damage, isolating bacteria from their environment, providing a carrier with a high probiotics load, allowing controlled and continuous probiotics release etc. In conclusion, it can be said that nano-encapsulation offers a promising outlook for incorporating live probiotic bacteria into foods and ensuring their survival during simulated gastric and intestinal processes. These findings provide valuable insights into the efficacy of encapsulated probiotics at different time intervals and support the need for further research in this area (Supplementary Information).

### Supplementary Information

Supplementary Information.

## Data Availability

The datasets used and/or analysed during the current study available from the corresponding author on reasonable request.
